# Improving the Lateral Resolution of Quartz Tuning Fork-Based Sensors in Liquid by Integrating Commercial AFM Tips into the Fiber End

**DOI:** 10.3390/s150101601

**Published:** 2015-01-14

**Authors:** Laura Gonzalez, David Martínez-Martín, Jorge Otero, Pedro José de Pablo, Manel Puig-Vidal, Julio Gómez-Herrero

**Affiliations:** 1 SIC-BIO, Bioelectronics and Nanobioengineering Group, Departament d'Electrònica, Universitat de Barcelona, Marti i Franques 1, 08028 Barcelona, Spain; E-Mails: laura.gonzalez.claramonte@gmail.com (L.G.); jordi.otero@gmail.com (J.O.); 2 Departamento de Física de la Materia Condensada, C-3, Universidad Autónoma de Madrid, Cantoblanco, 28049 Madrid, Spain; E-Mails: davidmmo@gmail.com (D.M.-M.); p.j.depablo@uam.es (P.J.P.); julio.gomez@uam.es (J.G.-H.)

**Keywords:** atomic force microscopy, scanning probe microscopy, quartz tuning fork, self-sensing probe, shear force microscopy, nanotip

## Abstract

The use of quartz tuning fork sensors as probes for scanning probe microscopy is growing in popularity. Working in shear mode, some methods achieve a lateral resolution comparable with that obtained with standard cantilevered probes, but only in experiments conducted in air or vacuum. Here, we report a method to produce and use commercial AFM tips in electrically driven quartz tuning fork sensors operating in shear mode in a liquid environment. The process is based on attaching a standard AFM tip to the end of a fiber probe which has previously been sharpened. Only the end of the probe is immersed in the buffer solution during imaging. The lateral resolution achieved is about 6 times higher than that of the etched microfiber on its own.

## 1. Introduction

In recent years, atomic force microscopy (AFM) based on a quartz tuning fork (QTF) has become increasingly popular, especially due to its self-sensing detection without the need for an optical feedback system. Furthermore, QTF-based sensors present low internal dissipation, high quality factors (*Q*) and high static spring constants (*K*), features that make these nanosensors very attractive for use in liquid environments to study biomaterials and biological samples [1,2]. Such properties allow stable low oscillation amplitudes to be achieved and thereby make it possible to work in a non-contact regime, avoiding the drawback of the tip to jump to contact at small tip-sample distances [3].

**Figure 1.**
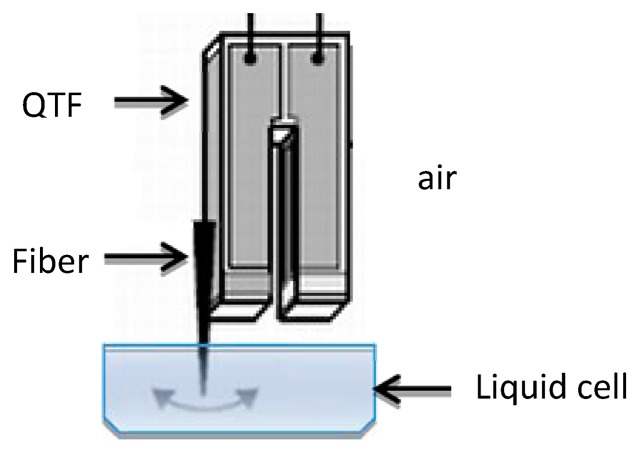
Schematic of the QTF configuration working in shear mode in a liquid environment.

There are two common QTF configurations, depending on which spatial direction the oscillation is in with respect to the sample surface: shear mode (parallel) or tapping mode (perpendicular). To work in liquid in shear mode, only the fiber end of the QTF sensor is immersed in the liquid; as illustrated in Figure 1.

QTF-based sensors are usually custom-built in the laboratory by manually attaching a sharpened fiber to one of the prongs of the QTF. The probe tips are generally made of silicon fiber that is tapered using chemical etching techniques or mechanically drawn using a commercial micropipette puller [4,5] to obtain a tip radius of 100–150 nm. The conventional etching method (Turner method) [6] consists of immersing the optical fiber in hydrofluoric acid with a layer of an organic solvent over it. At the interface between the two layers the meniscus height decreases and this is where the taper forms. This process has been refined to optimize probe properties such as surface roughness, cone angle and especially the tip geometry and radius. New techniques have also been appeared: tube-etching [7], selective etching [8] and dynamic meniscus etching [9].

Different setups and additional production steps have been devised to achieve a similar tip radius to that of commercial AFM tips; although it remains unclear how effective they are at imaging in liquid media in shear force mode. Some of the different techniques employed to reduce the radius in QTF probes are: etching different materials [10,11], incorporating microfabrication processes [12,13], integrating non-commercial tips made of different materials such as diamond [14] or attaching commercial AFM tips [15,16].

Different materials have been used as chemically or electrochemically sharpened probes for QTF sensors and they have been employed in different scanning probe microscopy (SPM) setups, such as near-field scanning optical microscopes (NSOM) and scanning tunneling microscope (STM)-AFM hybrid systems: nickel in [17], polymethylmethacrylate (PMMA) in [10] and carbon fiber tips in [11]. Recently, Jung and co-workers [18] proposed a method to produce polymer tips on the cross-section of optical fibers, capable of achieving a 45 nm radius tip. Other strategies based on different microfabrication processes have been devised to integrate a sharp tip into the resonator for use in tapping mode: lithography in [12], anisotropic wet etching and a focused ion beam (FIB) in [13] and the commercially available Akiyama probe [19,20].

Attaching commercial AFM tips at the end of one of the prongs of a QTF has led to the same apex radius as standard AFM tips. For tapping mode, [21] attaches silicon AFM tips to image biological samples and [15] shows atomic resolution and magnetic contrast. However, the application of these nanosensors is limited to air-based measurements. For shear mode, a cantilever segment is glued to the QTF for noncontact mode in air in [16,22] or for shear-mode magnetic force microscopy in [23].

However, studies in liquid environments in both tapping and shear mode require the immersion of the attached AFM tip in buffer solution. If a larger amount of buffer is used, the surface tension readily elevates the meniscus, thereby covering the electrodes and causing a short-circuit. Thus the external electrodes of the QTF preclude its utilization in aqueous solutions unless the whole sensor is coated with an electrophoretic paint [24]; but as the whole sensor is immersed in liquid, the Q factor decreases and sensitivity does too. This problem can be overcome by using such a small volume of buffer that it only just covers the surface of interest; although the drop of liquid evaporates rapidly. One way to resolve that problem is to use a liquid with a much lower evaporation rate than that of water-based buffers [25]; but then the sample will not be under physiological conditions.

In this work we present a feasible technique to improve the lateral resolution of a QTF in shear mode in a liquid medium, for studies of biological samples. Our method is based on attaching a standard AFM tip to the end of a fiber that has previously been sharpened chemically. As the QTF can be maintained oscillating in air while the tip is immersed in the buffer, our approach overcomes the main limitation of previous work in liquid environments.

## 2. Material and Methods

In our experiments we used QTFs with a resonance frequency of 32,768 Hz (AB38T model, AbraconCorp, Irvine, CA, USA). The dimensions of the devices are LxWxT = 4 × 1.25 × 0.4 mm and present a spring constant (*K*) of 34,000 N/m and a Q factor of 100,000 in vacuum. As a first step, the metallic cover of the resonator was removed and a chemically sharpened SiO_2_ fiber was attached to one of the prongs of the QTF. To taper the probes, a 125 μm optical fiber (SM600 single mode fiber from Thorlabs, Munich, Germany) was dipped into 40% HF solution with a layer of iso-octane (C_8_H_18_) on top to protect against the acid vapors, as proposed in [26]. The process is self-terminating and lasted around 90 min. Once the etching had stopped, the fibers were rinsed with ethanol and water and mounted onto the resonator with cyanoacrylate glue, with a length of 3–4 mm protruding from the fork. This is the minimum length necessary to work with the liquid cell while maintaining the QTF resonating in air. The tip radiuses were between 100 and 200 nm, but for our purpose this was not critical since we intended to reduce the fiber diameter until just enough surface remained to maintain contact between the AFM tip and the probe.

**Figure 2.**
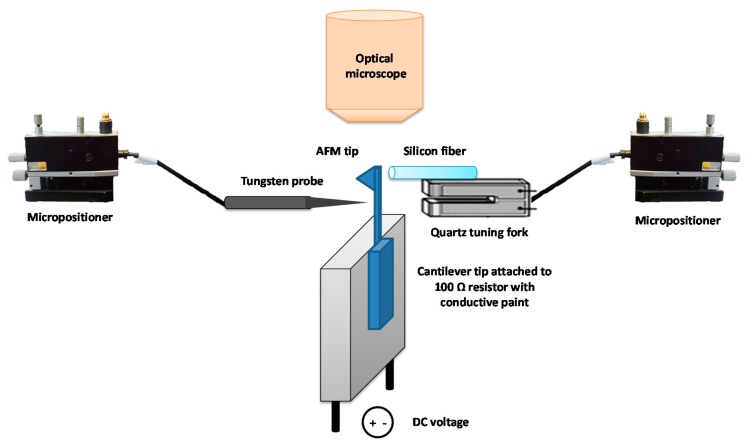
Schematic of the setup for the tip attachment procedure showing the resistor where the cantilever is glued with silver paint and the two micrometer manipulators: one for the tungsten tip and one for the QTF sensor with the fiber previously sharpened. The fiber end and cantilever tip are aligned under an optical microscope (10× Carl Zeiss lens). The objects in the figure are not to scale.

**Figure 3.**
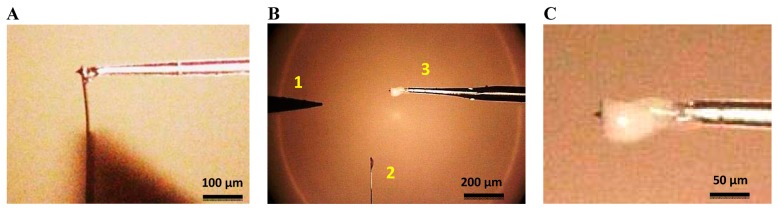
(**A**) AFM cantilever tip glued to a silicon fiber previously sharpened by chemical etching; (**B**) A tungsten tip (1) is employed to break the leftover segment of the cantilever (2) when the glue is dry; (**C**) Magnification of image B showing the AFM tip attached to the fiber probe (3).

Once the fiber was mounted onto the QTF, the sensor was placed in a micropositioner with a magnetic base incorporating 3 DOF of movement (from SUSS Microtec, Munich, Germany). The cantilever chip was attached with silver paint to a 100 Ohm 30 W 5% resistor, with the cantilever protruding from the resistor. Figure 2 is a schematic representation of the setup for the tip attachment procedure. The cantilever and QTF probe were aligned under an optical microscope with a 10× objective (Zeiss EC EPIPLAN, NA = 0.25). The fiber was placed at the very end of the cantilever on the opposite side from the tip (see Figure 3A). The tips used in this work were commercial chips (CONT model, NanoWorld, Neuchâtel, Switzerland). The AFM probes were made of Si without any coating, with 450 μm cantilevers and a nominal spring constant of 0.2 N/m. The tips were 10 μm high and presented a nominal radius of 10 nm.

We used Nural 26 glue (Henkel Ibérica S.A., Barcelona, Spain), as it has strong adhesive properties (100 kg/cm^2^ after 6 h), to attach the AFM tip to the fiber. We were able to accelerate the hardening by increasing the temperature to approximately 70 °C. We needed rapid drying because the longer it takes; the more easily the fiber becomes misaligned from the AFM tip. Thus, the resistor terminals where DC polarized and the heat of the resistor dissipated accelerating the drying process. A temperature of 85 °C was reached in the resistor at 15 V. As the amount of glue needed was very small, the hardening took around 90 min.

After attaching the fiber to the cantilever, we removed the cantilever from the glued tip using a tungsten probe (model 17680 from Cascade Microtech GmbH, Dresden, Germany) placed in another micropositioner. We also removed the leftover segment of the cantilever. The resistor to which the AFM chip was connected was then grounded and the STM probe connected to a DC voltage. The STM probe was moved close to the cantilever just below the tip, a voltage of 15–20 V was supplied to create a short-circuit and the cantilever was broken off at the point of contact, as illustrated in Figure 3B. The most critical step was the last one: placing the tungsten tip in the correct position, close enough to the cantilever to break it but without damaging the tip. Even so, the process was successful around 80% of the time.

The sensor was mounted on an adapted STM head integrated into a commercial AFM microscope (Nanotec Electrónica, Madrid, Spain). A fully custom-made electronics module was used to drive the QTF electrically [27] to its resonance frequency. A transimpedance amplifier (TIA) (model OPA656, Texas Instruments, Dallas, TX, USA) converted the current through the QTF into voltage with a 10^6^ V/A gain. The current losses due to parasitic capacitance related to the contacts and cables were compensated with a subcircuit with the same capacitance but 180° phase shifted [28]. A driving signal was applied to drive the QTF at its resonance frequency using an integrated generator in the lock-in amplifier. The final resonance frequency after adding the extra mass of the fiber and AFM tip was shifted to 32,720 Hz and the resulting Q factor was 2647.

## 3. Results and Discussion

### 3.1. Measurements on Calibration Grid

To evaluate the lateral resolution, a silicon test structure with a pyramidal calibration pattern was scanned (model TGG01, Mikromasch, Sofia, Bulgaria). The image was registered in amplitude modulation where the input of the main feedback is the amplitude of the QTF current signal, while the variable phase is also recorded. We employed a low voltage-driven amplitude (3 mV) to minimize the amplitude of oscillation of the probe and reduce the effective tip radius. A vibration amplitude of ∼1 nm was derived as reported in [29]. The set point of the feedback was set to 83% of the maximum free amplitude at the resonance frequency: *A_set_*/*A_free_* = 0.83.

**Figure 4.**
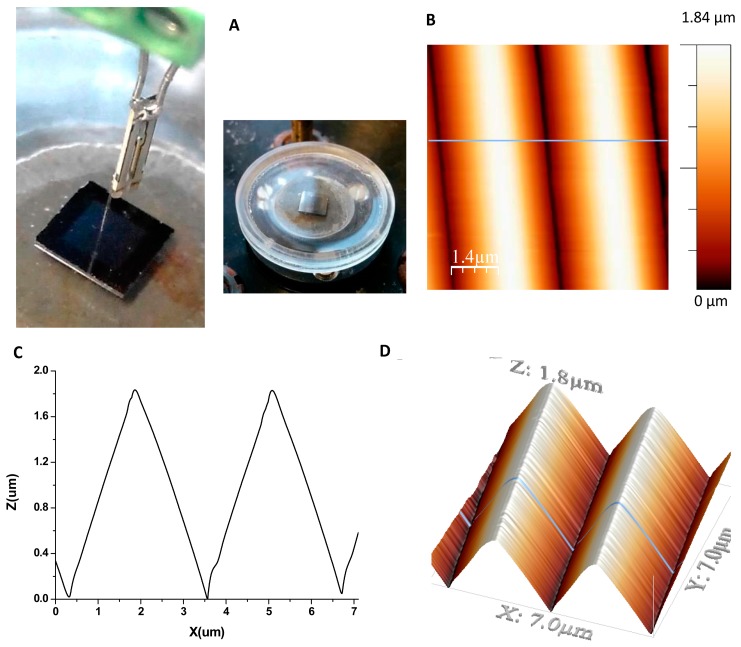
(**A**) Measurement set-up. The QTF probe is immersed in buffer solution to access the sample using a liquid cell; (**B**) Shear force image of the TGG01 3 μm pyramidal calibration grating. Image size is 7 μm × 7 μm; The topographic profile of 1024 p. and the same image rendered in 3D are shown in (**C**) and (**D**) respectively.

The calibration grating is characterized by the small curvature of the edges (less than 10 nm) and it has a pitch of 3 μm and a height of 1.8 μm. The fiber probe with the AFM tip at the end was immersed in the buffer solution where the sample was also submerged into the liquid cell (see Figure 4A). Figure 4B,C are images of the topography and profile, respectively, of the grating taken in shear force mode with an AFM tip mounted on a QTF fiber probe in buffer solution using a liquid cell. The measurement buffer was PBS with a pH of 7.4. As shown in Figure 4D, the scanning area was 7 μm × 7 μm and the line scan speed was 0.28 line/s.

**Figure 5.**
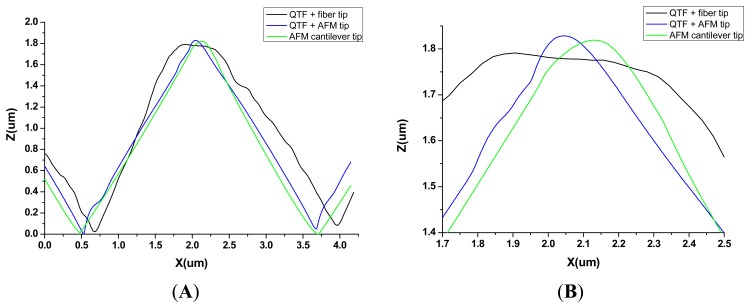
(**A**) Direct comparison of the topographic profiles of one of the features of the imaged calibration pattern with: QTF with fiber, QTF with AFM tip and with AFM cantilever tip; (**B**) Magnified image of the edge of the pyramidal structure. Notice that about 6 blue peaks fit within the black naked fiber profile.

The resulting shape of the edges of the pyramids in the image is the dilation geometry of the tip and the pattern [30] and it is difficult to quantify the tip radius exactly, as the two geometries are unknown. Since the performance of the tip is related to the sharpness and acuteness of the line profile [31], we imaged the same structure in a buffer solution with a QTF-based sensor with a fiber probe, and with an AFM cantilever tip to compare the topographic profiles. The results are shown in Figure 5, where the topographic profile of the previous image taken with the AFM tip attached to a QTF (blue line) is superimposed over the profiles obtained with the other two sensors: QTF with a fiber probe chemically tapered (black line) and a commercial AFM cantilever tip in tapping mode (green line). In general terms, the profile obtained from the image taken with the QTF with a fiber probe is of poor quality, and it is clearly distorted by the irregular shape of the fiber. The improvement in lateral resolution of the tip apex through adding a commercial AFM tip to the fiber probe (blue, Figure 5) is approximately 6-fold, compared to that of the naked fiber (black, Figure 5). In contrast, similar image quality was obtained with the AFM tip attached to the QTF and with the commercial tip, as expected. Both are capable of handling large variations in sample height and show well-defined edges. Qualitatively, the AFM cantilever tip represents the triangular shape of the pattern slightly better with smoother slopes. This could be due to the effect of the lateral oscillation of the tip when working in shear mode with the QTF. Even though the driving amplitude is very small (3 mV) to minimize the lateral oscillation and it is damped by the viscosity of the buffer, lateral resolution could be affected by an extremely low increase of the effective tip radius. Nevertheless, we cannot appreciate any significant difference between the two profiles amplified in Figure 5B.

### 3.2. Measurements on Soft Samples

Once the resolution is determined with a calibration grid, a soft sample is imaged in liquid in order to check the feasibility of the developed technique. The sample contained *Escherichia coli* bacteria in stationary phase. For sample preparation, the sterile loop was used to take a small quantity of bacteria from the agar plate into 10 mL of LB and it was left at 37 °C at 250 rpm for 15 h. 600 μL were centrifuged at 3000 rpm for 3 min. The pellet was re-suspended in other 600 μL in Milli-q water diluted 1/20. A drop of 40 μL was pipetted onto the freshly cleaved HOPG substrate. Then, the sample was left to dry for 1 h to improve the fixation to the substrate and then rehydrated with PBS solution to be placed in the fluid cell and imaged with the tuning fork sensor.

**Figure 6.**
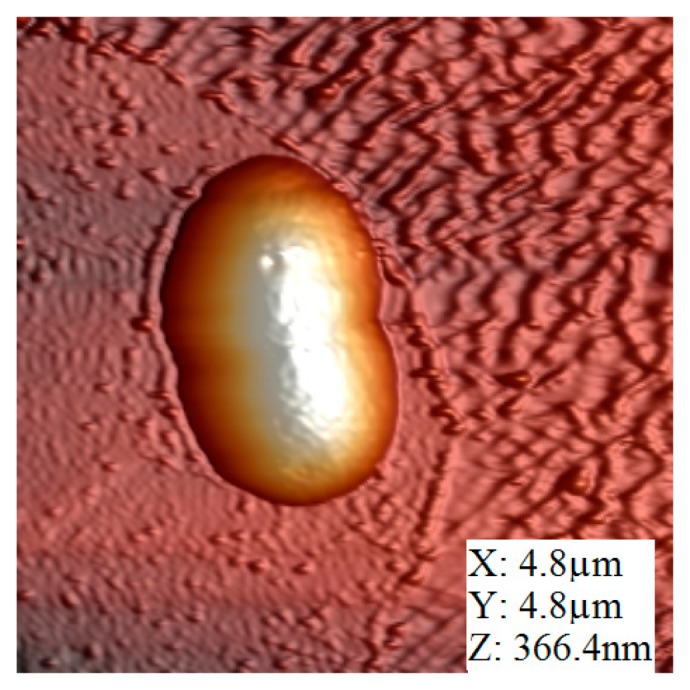
Shear force image of a single *E. coli* bacterium. Image size is 4.8 μm × 4.8 μm.

The image was also taken in amplitude modulation applying low driven voltage to minimize the oscillation amplitude. The set point of the feedback was set in this case to 87% of the maximum free amplitude at the resonance frequency. As shown in Figure 6, the scanning area was 4.8 μm × 4.8 μm and the line scan speed was 0.5 line/s. Compared, with the silicon test structure, in this case the tip-sample interaction must be gentler avoiding sample damage while scanning.

## 4. Conclusions

Here we report the first feasible technique to obtain a QTF-based sensor with the same tip radius as commercial AFM tips, to work in shear mode in liquid environments. Previous works have demonstrated that the attachment of commercially available AFM tips increases lateral resolution. Nonetheless, in previous research the AFM tips were directly attached to the end of one of the prongs of the tuning fork. Therefore, to use the QTF sensor to take measurements in liquid environments at least part of the prong needed to be immersed in the buffer solution (damping the oscillation of the QTF and therefore reducing its quality factor). Moreover, if the liquid is conductive, the electrodes of the nanosensors might be short-circuited. The work presented here (attaching the AFM tip to the end of the silicon fiber tip) resolves these issues. The method for preparing and joining the AFM tips to the QTF is easy and reliable. We notably improve the tip apex of the probe compared to the chemically sharpened fiber, achieving the same performance as that of a commercial AFM tip. A comparable topographic image of a specific patterned structure and a soft sample are successfully obtained in a buffer solution. We show that with our sensors it is possible to acquire high resolution images in liquid media, necessary to study biomaterials or biological samples under physiological conditions. This process overcomes one of the limitations present in shear force microscopy using QTF-based nanosensors to work in liquid media as the lateral resolution is equivalent to that achieved with a commercial AFM tip.
